# Supplementary material for: PTEN Protein Phosphatase Activity Is Not Required for Tumour Suppression in the Mouse Prostate
Source: Biomolecules. 2022 Oct 19;12(10):1511. doi: 10.3390/biom12101511 (PMC9599176; doi:10.3390/biom12101511)
Supplement: Supplementary file 1 [file biomolecules-12-01511-s001.zip › Supplementary Figures and Tables.pdf]

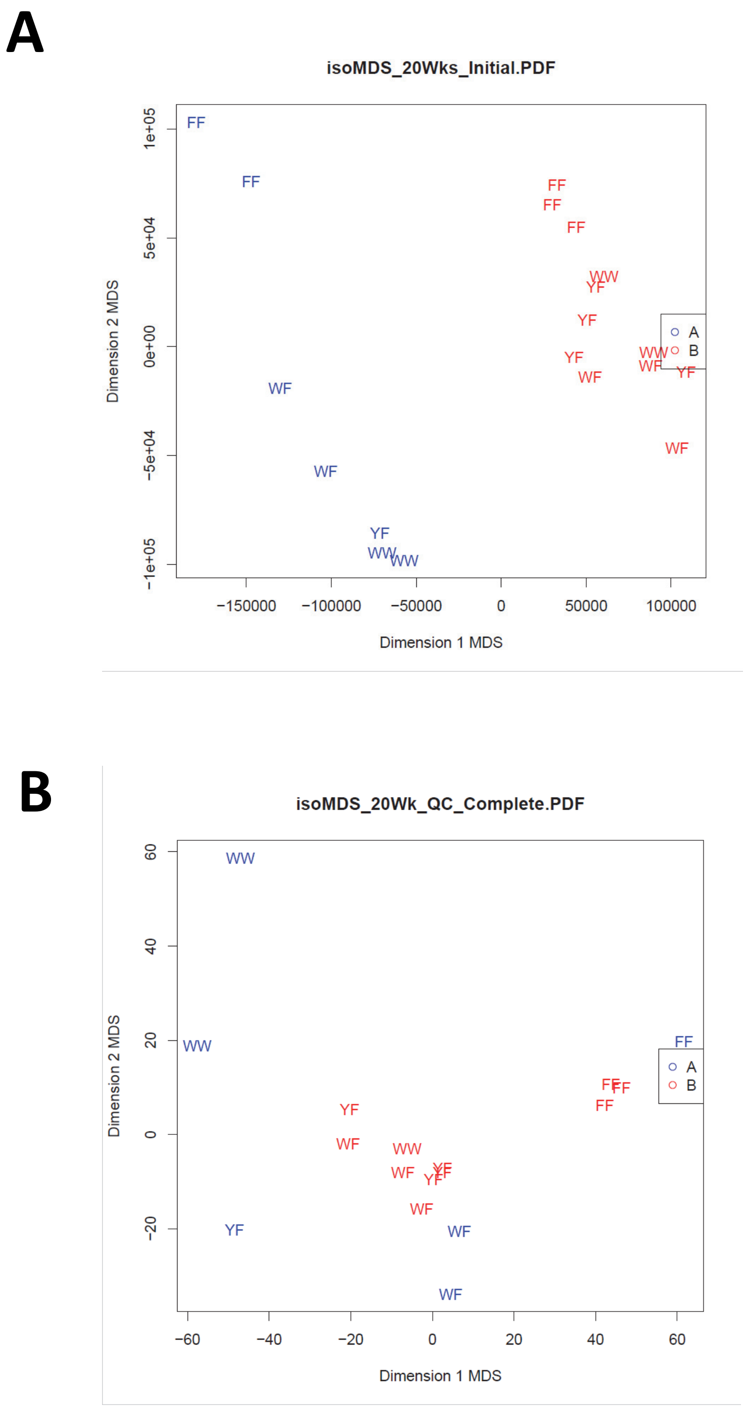

Supplementary Figure S1. A. Initial multidimensional scaling plot, showing sample clustering for the 20 week samples. A clear batch effect was observed separating batch A (blue) and batch B (red) samples. **B.** Multidimensional scaling plot following completion of QC; including batch correction, quality filtering, background correction, aggregation of probes into probesets, and quantile normalisation. Batch A is shown is blue, batch B in red. The batch effect has been mitigated and biological variation indicated by sample labels (WW, WF, YF, FF etc.) is the major factor driving the location of samples in the plot.

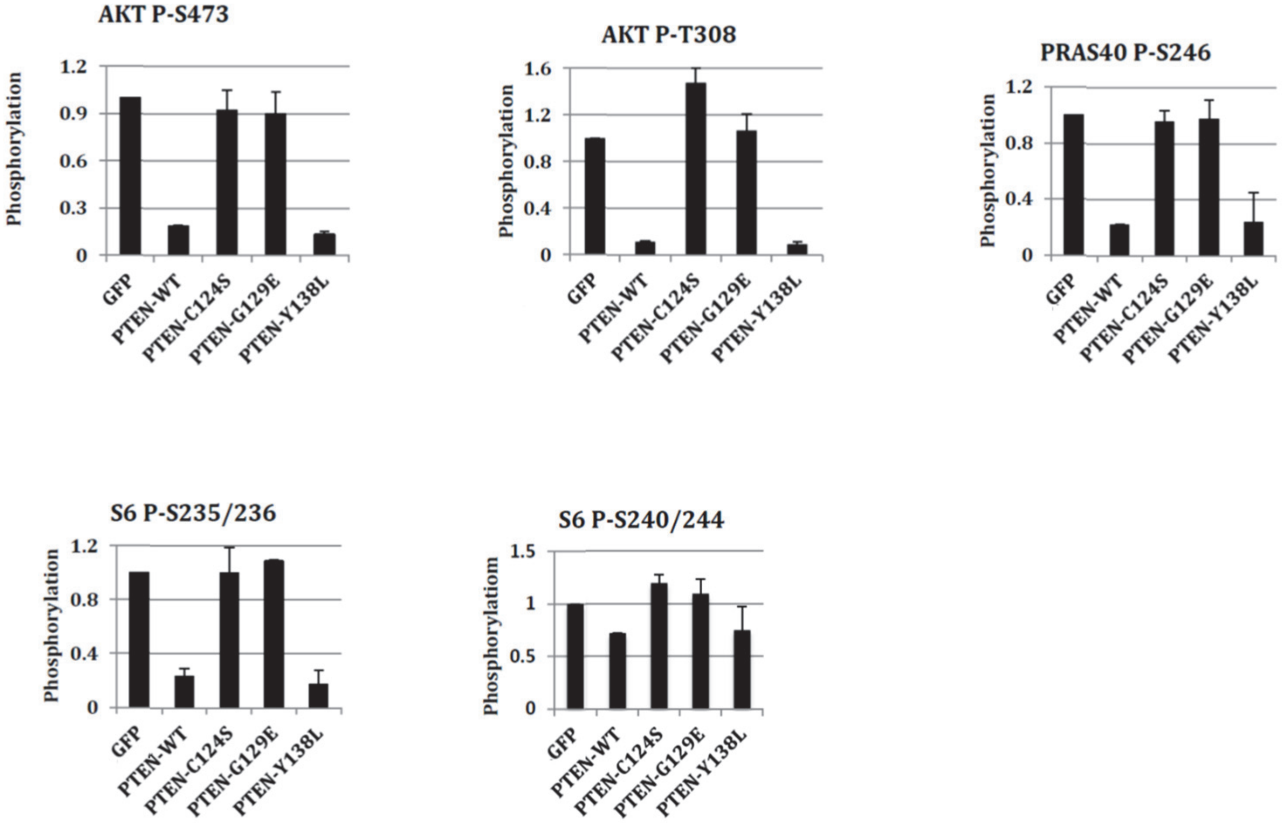

Supplementary Figure S2. Quantitation of immunoblotting data from LNCaP cells expressing PTEN. The shows mean quantitation from two independent experiments, one of which is shown in Fig 1A. Signals were captured and quantified directly by CCD camera imaging of immunoblot luminescence. Values from GFP expressing control cells in each experiment were set at 1 and other relative mean values calculated and presented.

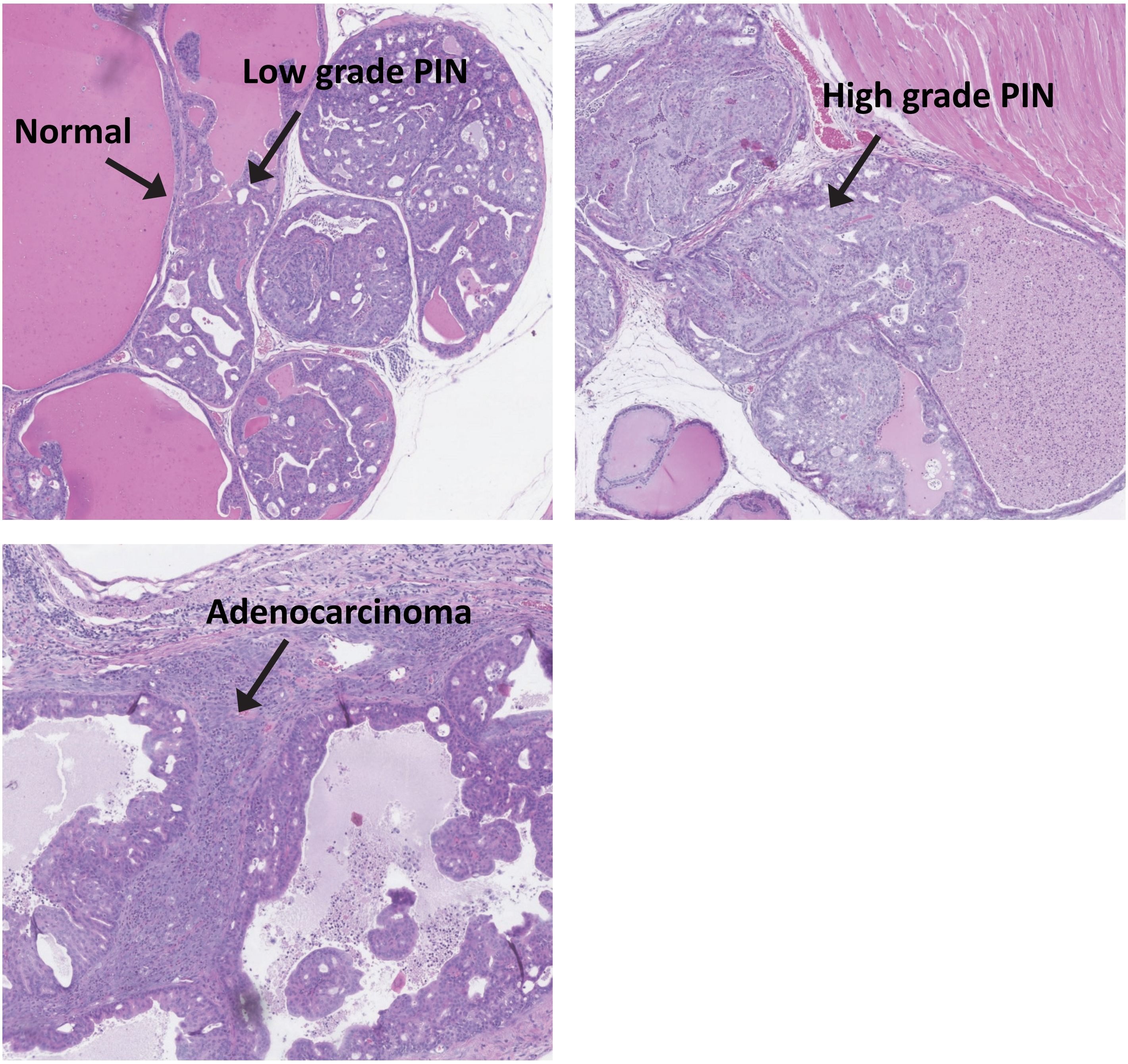

Supplementary Figure S3. Illustrative examples of low grade prostatic intraepithelial neoplasia (PIN), high grade PIN and prostatic adenocarcinoma in *Pten$^{fl/fl}$ Pb4-Cre* prostates are shown.

**All Data:** 6 Week Samples, (FE > 1.3)	WF vs FF		WF vs YF		WW vs FF		WW vs WF		WW vs YF		YF vs FF	
Functional Category	***WF Upreg***	***FF Upreg***	***WF Upreg***	***YF Upreg***	***WW Upreg***	***FF Upreg***	***WW Upreg***	***WF Upreg***	***WW Upreg***	***YF Upreg***	***YF Upreg***	***FF Upreg***
Annexin/Phospholipid-binding		3.36		3.84		3.38						2.16
Antiviral Enzymes (oligoadenylate synthetase)		2.33				2.25						2.26
Cadherin/Cell-Cell adhesion		1.81				1.84				1.52		
Carbohydrate binding/Lectin										1.69		
Cell differentiation/development		1.71				1.43						1.45
Cell membrane anchoring /glycoproteins		2.76				2.79						3.02
Cell structural changes				2.04		1.78						1.79
Cell surface/negative apoptotic regulation/protein complex										1.90		
Disulfide bond/ signal peptide/ Glycoprotein		6.96		3.08		6.55	2.06			2.09		6.44
Histones/DNA Binding		2.10				2.13						2.22
Innate immunity/inflammatory response		10.53		1.43		10.62						10.69
Integrin/ ECM binding		1.72				1.74						1.77
Netrin domain/ metalloproteinase inhibitors		1.77				1.79						1.79
Scavenger receptor activity						1.66	2.82					1.66
Serpin protease inhibitors		1.46		1.45		1.49						1.50
Signal peptide/extracellular secretion	2.10	5.84			2.49	5.40			1.92		2.53	5.41
Transmembrane cellular transport/ glycosylation/ glycoprotein		2.49				2.45						
Viral defense response		2.08				2.10						2.11
Viral response pathways		1.55				1.57						1.60

Supplementary Table S1: Significant Enriched Annotation Clusters, All Genotypes 6 weeks. Results reflect clustering of enrichment terms followed by calculation of a significance for the entire cluster of similar terms. The groupings below show the significant annotation clusters that were upregulated between each genotype pair

	Number of genes differentially expressed in all studies (F/F relative to W/W)	
Mouse ages in studies	Higher	Lower
6-12 Weeks	34	9
15-30 weeks	49	33

Supplementary Table S4. This shows the numbers of genes found to be differentially expressed in *Pten* null prostate tissue relative to wild-type samples in the gene expression data described here and also in all 3 comparable published datasets relevant to each age group. 6 external datasets were compared to the 2 datasets derived in this study. The inclusion threshold was a difference in log fold change of >1.5 and p<0.05. W/W = $Pten^{+/+}$; F/F = $Pten^{flox/flox}$. The genes are identified and listed in Supplementary Table S5.
